# Miscellaneous

**Published:** 1874-05

**Authors:** 


### MISCELLANEOUS.

### HEALTH RESORTS.

### Watkins Glen and Magnetic Springs.

Watkins is located at the head of Seneca Lake. The village contains about three thousand inhabitants. Passing up the Main street, south and parallel with the mountain slope, a walk of half a mile brings one to a bridge which spans the stream that flows through the "Glen."

The entrance to the "Glen" is about five hundred feet west of this bridge. The stream has cut its way into the slope of the mountain for over two miles, forming cavernous recesses, ravines, waterfalls, foaming rapids and deep pools.

The mode of ingress for visitors is by rude stairways, running diagonally along the face of the wall. Landing places are provided at intervals, from which other stairways spring; and thus the ascent is made, and the entrance to the gorge is surmounted.

We now enter "Glen Alpha." Inside the great rock barrier, which we have just passed, a bridge spans the chasm, and from this a fine view is obtained of the first cascade, as it falls into a deep basin scooped out of the solid rock by the constant chafing of the swift waters. Leaving the bridge and mounting up a series of steps, we presently reach a narrow foot path, cut out of the face of the cliff, and follow its windings until further progress is barred by a transverse wall, over which the waters of the long cascade fall from a great height into the pool below. At this point the rugged and lofty walls of the gorge draw close together. Where the foot path ends, a long staircase, wet with the mist and spray of the cascade, is flung at an angle of ninety degrees across the chasm, and at its upper end connects with another foot path, about fifty feet above the one just left. After following this path a little distance, we come to a series of cascades, dropping from one low ledge to another, with deep pools and broad shallows intervening.

Pursuing our onward and upward course, the rocks take on more grotesque forms, and the air is moist and cold. The path —a mere ledge in the face of the cliffs—overhangs a deep chasm, at the bottom of which the waters foam and gurgle. Overhead the gray walls rise, tier upon tier, inclining gradually toward each other, until only a strip of sky can be seen, with the light struggling dimly through a fringe of hemlocks. Beyond this gloomy pass, the ledge we are traversing ends abruptly, and the obstacles to farther advance have to be overcome by a succession of stairways, now crossing to one side, now changing to the other, until, by ever-ascending grade, another pathway is reached. Here the rock walls recede, and sufficient soil has accumulated over them to admit of the growth of shrubs and large evergreen trees. The path, too, is easier. Following it for a short distance, we come to a stairway placed against the bank, and, on ascending it, reach a shelf of the mountain on the north side of the ravine.

On this shelf is the Mountain House, built somewhat after the style of a Swiss *chalet.*

Leaving the Mountain House, the path dips steadily downward, almost to the bed of the stream ; and, after passing another series of small cascades and rapids, we cross a bridge to the opposite side of the gorge, where the cliffs, rent and torn into every conceivable shape, first contract and then expand into an enormous amphitheatre, to which has been given the name of Glen Cathedral. The area is vast. The immense walls, nearly circular in form, rise to a great height, and, where they terminate skyward, are crowned with the fresh, green foliage of the hemlock. The floor of this amphitheatre is almost as level as if it had been paved by human hands ; and over the great slabs of rock, laid regularly and closely jointed, the stream spreads out, but an inch or two in depth, flowing easily and quietly, with scarcely a ripple to break the smoothness of its surface. Passing through a break in the great circular wall, by a path still broad, but more broken and water-worn, the tall cliffs recede upward from their base ; and on the slopes thus formed and shelving outward, some hemlocks and deciduous trees find sustenance. Suddenly, the tall cliffs, as if spurning these picturesque accessories, close in again, and in the cavernous gloom of the remote distance another cascade is seen flowing in a white sheet over its rocky ledge, and pouring its waters into the gorge. On nearing this fine cascade, another stairway, thrown across the gorge to a higher shelf projecting from the face of the cliff, gives access to a remarkable scene. Before us is what is called the Glen of Pools, from the variety and extent of its water-worn basins. Standing on the bridge and looking up the gorge, the eye falls upon a series of cascades and rapids, low and broad, but very beautiful. The enclosing walls are again sufficiently broken to allow of the growth of trees in some places, and to let the light in freely. Beyond these, again, cascades of greater breadth drop from one rocky ledge to another, foaming and seething, while over the southern wall, and the pathway that clings to it, a thin stream, falling from a great height, spreads itself out like a veil of silver mist, and mingles its waters with those in the rock-bound channel far below. At certain seasons of the year the sun is at an angle which sends glancing lights through the gorge, which break in prismatic colors on this thin fringe of a waterfall, and hence give it the name of Rainbow Falls. But the nomenclature of the Glen is hopelessly free and confusing, each season giving a new series of designations to its various falls and aspects.

(*To be continued in June number.*)

### STAR LECTURING.

Mr. Proctor does not need to look upward to find the star depths. The phrase may fitly characterize American society, which consists of stars and blank spaces. We run our politics on the starring system. A man becomes a star and we make him president. The "red light of Mars" is the favorite color. Not statesmanship, not personal character, not intellectual culture, not eminent knowledge, not anything and not any combination of things that constitute superlative fitness, fixes the American choice for the chief magistracy. The star which, for the moment, can attract the greatest number of eyes, becomes the lord of the heavens and the earth. Votes must be had at any sacrifice; and votes can only be counted on for stars. Availability is the political watchword, and such statesmanship as we get is that with which we manage to surround the star that so quickly cools and flickers in its new and alien atmosphere. Political rewards do not go where they belong; public trust is not reposed in the best men; and so politics degenerate, and second and third rate men are everywhere uppermost. The starring system in politics is a failure. It is bad for the country; it is bad for politics; it is a discouragement to personal and political worth; it is a nuisance.

The starring system in theatricals is even more obviously destructive to all that is worthy in the popular drama. We go to a theatre, not to witness a play, but to see Booth, or Joe Jefferson, or some other star. The opera is nothing without Kellogg, or Patti, or Nilsson, or some miraculous tenor who to-day is and to-morrow is not. The orchestras—trained, laborious, patient, admirable—pass for nothing. The choruses are not thought so much of as an orchestrion would be. The great mass of singers and players who sustain the minor parts have no more consideration than puppets. What is the consequence? The money is mainly absorbed by the stars, who shine the brighter in a sky of mediocrity or absolute inferiority. So long as the starring system prevails, mediocrity will be the rule. Stars must have space to be seen; and we have had for years, in the theatrical world, nothing but stars and spaces—the latter wide. A first class drama, well presented in every part, has not been witnessed in New York for a long time; and for this fact the starring system is alone responsible. An actor now-a-days can get no consideration except as a star, and, to succeed, he is often obliged to confine himself to a single play.

How has the starring system worked upon the platform? It has been tried pretty thoroughly for the last five years, and the results ought to be, and are, apparent. Ten and fifteen years ago, a course of lectures consisted of eight or ten discourses on topics of popular interest, or social and political questions of public moment. They were prosperous, well attended and profitable in many ways. Then came the star fever. Men were summoned to the platform simply because they would draw, and not because the people expected instruction or inspiration from them. A notoriety had only to rise to be summoned at once to the platform. If he could lift a great many kegs of nails; if he was successful as a showman; if he was a literary buffoon, and sufficiently expert in cheap orthography; in short, if he had been anything, or done anything to make himself an object of curiosity to the crowd, he was regarded as a star, and called at once into the lecture field, for the single purpose of swelling the receipts at the door. Of course, the stars called for high prices, and under high prices the number of lectures given in a course was cut down. The people who came to bask in the blaze, finding too often only a twinkle, and sometimes only a fizzle, that left an unpleasant odor, became disgusted, and the best of them—the very men and women upon whom the whole lecture system relied for steady prosperity—left the lecture room altogether. Still the starring system went on, with a new agency to push it, established by the lecture bureaus. Men were invited to come from England, and promised great results. Some of these have been genuine accessions to the corps of good lecturers, while many have proved to be sorry failures. Many a famous name, "far fetched and dear bought," has shone upon the list for a season, never to be recalled and always to be remembered with disappointment. The bureaus have pushed and puffed their pets—both imported and domestic—until lecture committees have ceased to believe in them altogether.

And now, what is the condition of the platform? In the large towns, where they have been able to get "the stars," it is difficult to get a first rate audience together on any night, and still more difficult to maintain a steady, prosperous course of lectures. In the smaller towns, where want of funds has compelled them to dispense with the stars, the system was never more prosperous than it is to-day. In New England and New York, generally, the towns with 20,000 inhabitants and upwards, have difficulty in sustaining a course of lectures, while there are many towns of less than five thousand people that maintain a good course every winter, and make money by it.

If there is anything in the lecture system worth saving, let us save it. Those who know what it used to be will be glad to see it restored to its old position; and if they have studied its history they will conclude with us that the starring system must be stopped. The lecture room must cease to be the show room of fresh notorieties, at high prices. Men must be called to lecture for the simple reason that they have something to say. The courses must be lengthened, and made in themselves valuable. The pushing by interested bureaus of untried men must be ignored or resisted. Men must be called to teach because they can teach, and not because they can do something else. The lecture must cease to be regarded simply as an entertainment. Wherever it has been so regarded and so managed, the system has gone down, and wherever the stock lecturer has been sacrificed to the star, the audiences have gradually dwindled until it has become almost impossible to sustain a course of lectures at all. Stars have been so much in fashion that we have establishments now for the manufacture of fictitious reputations, and these establishments must go under. They always were an impertinence, and they have become a nuisance. The lecture is a necessity. Let us restore the institution to its old footing of direct friendly relations between the lecturers and the lyceum, and give no man access to the platform who does not come there in a legitimate way, and who is not held there because he has something valuable to say. No system can stand when its best and most reliable workers are pinched in their prices, that those may be overpaid who not only bring no strength to it, but weaken it in its finances and in its hold upon the respect and affection of the people.—*Dr. J. G. Holland, in "Scribner's" for May.*

### POLITICAL MORALITY.

We hear a great deal now-a-days about the rule of "second rate men;" and there are many good people who fancy that the country is suffering from the lack of great statesmanship among our law makers and the executive officers of the government. There is a measure of justice in this judgment, without doubt, but it does not cover the ground. There is, at least, ability enough among these men to carry their policy, whatever it may be. There is no lack of ingenious subterfuge, far reaching intrigue, bold and powerful leading, and personal influence and public eloquence to compass any end desired. There is no lack of instrumentalities to push any approved party scheme; to forward any special interest; to advance any sectional policy; to secure the personal aggrandizement of any pet of a cabal. From the low standpoint of the prevalent political morality there seems to be abundant intellectual ability to carry any desirable measure. It is not the brains that are at fault; it is the heart. It is not ability that is wanting; it is morality.

Have we at the head of the government a man of high toned morality?—a man whose supreme desire is to do right?—who, above all personal interest, above all party policy, above all the influence of corrupt men, is exercised by the dominant purpose to keep his conscience clear and his hands clean, and to serve his people with unswerving integrity? Are the leaders of our national councils and the men of influence there trustworthy men? Are they men who take the straight path of duty and follow it, irrespective of all the bribes which power and wealth hold in their hands, and regardless of all the threats of unprincipled bullies and intriguers? We are not called upon to answer these questions; but all those who feel compelled to give them a negative reply hold in their hands a sufficient explanation of the evils from which the country suffers to-day. It is not necessary to point to the *Credit Mobilier*, or the Salary Grab, or any of the corrupt schemes by which these men betray themselves. A low morality among our legislators and rulers gives all through, by a fatal necessity, immoral legislation and immoral administration. If this low morality exists, every interest of this great country is at its mercy. All the national questions that arise must be settled by it. It vitiates the national policy; it poisons every political measure; it narrows everything down to the limit of its own party and personal interests.

The people of America richly deserve the infliction of all the evils from which they suffer. From the time of General Jackson to this day there has been practically but one rule in the selection of a chief magistrate, and that rule has been mainly followed in the election of our national legislators. This rule is known as "availability." Each party has put forward for its leaders, little and large, those who for any reason seemed likely to get the greatest number of votes. In no instance during this period have we had in the presidential chair a first class man. Some of our presidents have been good, but weak; some have been old political trimmers; some have been boors who were the laughing stock of the nations; some have been mulish and ignorant. Certainly no one has stood upon an equality with Washington, Jefferson and the Adamses. The question of morality is not one with which our people have concerned themselves at all. Men have been chosen because they were not statesmen; because they were unknown; because they were popular with the rabble; because they were good soldiers; because they could get votes. We do not know of one man during the whole period who has been chosen because he was a Christian gentleman and statesman, and so above all unworthy motives in the administration of the duties of his office. Of the rules that prevail in the election of our national legislators our readers are good judges, and they know that almost everything else is considered before morality. There are men of influence in both houses of Congress, whose personal characters and histories will not bear inspection for a moment—men with whom no one can come into association without a stain.

Far be it from us to deny the presence of good men in Congress. There are as noble men there to-day as there ever were, but their influence is nullified by their bad and unscrupulous associates. Some of the very men who are trusted least by the country, and whose moral reputation is a stench in the nostrils of the world, are most prominent in the national councils and most powerful in the direction of government patronage.

We long ago ceased to expect perfection in the world of politics; but the duty of every honest man to try for it never ceases. When we get honest men in the places of trust—men with whom honor is more than money, and duty more than preferment, and country more than party, and God more than all—we shall have wisdom in law and purity of administration. Personal immorality and wise statesmanship cannot exist together, and until the American people insist that their public servants shall be gentlemen, at the least, they must expect to suffer at large from the conflicting policies of selfish and corrupt men.—*Dr. J. G. Holland, in "Scribner's" for May.*

### HUMBOLDT.

A CURIOUS note, which comes from Russia, is of a visit which Humboldt once paid to Iszym in Siberia. Carrying a letter of introduction from the Governor-General, he went to the house of the chief government functionary of the place, M. Skotin, who was apparently a sort of Goodman Dull of immense self-importance and ignorance. The first thing that the Governor-General heard of the *savant* was in a long letter from the wise Skotin:

"Some days ago," he said, "there came here a German named Gumboldt, a dried-up little man, looking anything but respectable. As, however, he brought with him a letter from your Excellency, in which I am directed to treat him with politeness and consideration, I received him with all due respect. At the same time, I must observe that this individual seems to me very suspicious, and even dangerous. From the first he did not please me; he gossiped too much, and did not like the fare I offered him, though I have a cook, Ferlisa, who makes excellent pirogs, and would be most happy to offer some to your Excellency. * * * He seemed to despise both myself and my hospitality, and he evidently looked down upon the most eminent officials of the town. On the other hand, he is continually talking with the Poles and other political criminals under my charge. Your Excellency will forgive my boldness in saying this, but these conversations with political criminals could not escape my observation, especially as a few days since, after a long conference, he went out with them at night to the top of a hill which commands the town. There they took out of a case which they had brought with them an instrument in the shape of a long tube, which seemed to me and my colleagues like a huge cannon. This they placed on a three-legged stand, and then aimed it straight at the town. Each of them approached the instrument, apparently to adjust it so as to rectify the aim. Seeing the great danger which threatened the inhabitants of the town, which is built entirely of wood, I immediately ordered the town guard, which consists of a sub-officer and six men, to march to the spot with loaded muskets and not lose sight of this German's proceedings. If the man's treacherous designs prove what I suspect them to be, we are ready to risk our lives for the Czar and for holy Russia."

### GOLD.

A day of bright reflections on the pond
And wavering shadows over moss and frond:
A wayward breeze, the summer's latest born,
Teased the stiff grain, and bent the stately corn,
Or rocked the bird-nests in the prickly thorn.

Above, the lavish sun filled air with gold;
Again, below, on mimic waves it rolled,
And hid in lily cups. Her netted hair
Gleamed in the splendor, bright beyond compare,
Forming about her head a nimbus rare.

The velvet mullen raised its yellow head,
The buttercups like precious ore were spread;
Like golden shuttles hung by spirit hands,
Weaving invisible their magic strands,
Darted quick oriols in joyous bands.

Fond helianthus turned her fervent face,
Meek antirrhinum paled and grew apace;
Late dandelions, robed in cloth of gold,
With golden-rod upsprung from out the mould,
And pensive, gold-eyed daisies pranked the wold.

As snowy, gold-rimmed cloudlets bide the sky,
So hide her eyelid's golden fringe her eye ;
As every growing beauty of the earth
But figures forth great Nature's hidden worth,
So my love's charms from her pure heart had birth.

Pure heart of gold to me that day was given,
And promise true as gold made earth a heaven:
Then far away fled every doubt forlorn ;
We felt for us the Golden Age reborn,
And envied none their gold from labor torn.

*Lippincott's Magazine.*

### WHERE IS SCANDINAVIA?

SCANDINAVIA is not a country to which a letter can be addressed; it is only an idea. But in history the ideas are of two kinds. The ex-King of Hanover may truly speak of his kingdom as an idea. But Hanover is an idea that has been ; Scandinavia is an idea that shall be. The one has died, the other is about to be born ; and while no one cares about the former, except the historian and, of course, the ex-King, every man who wishes to know a little more of what is going on in the world than what he can see from his own window, may take an interest in the latter.

The Scandinavian idea means a combination of the three northern countries—Sweden, Norway and Denmark—into one country, Scandinavia. It intends to make the three different nations which inhabit these countries one people, and the three different States which these nations have established, one political body. It aspires to be the inaugurator of a new people and the founder of a new State. It is an idea of great pretensions and golden promises.—*Clemens Petersen, in "The Galaxy" for May.*

### THE AMERICAN GIRL ABROAD.

TO THE French matron this girl is an enigma. Where her daughter, timidly and with downcast eyes, answers the man with furtive speech, her sister from over the sea confronts him boldly and speaks with assurance. One blushes when she is accosted by the man, while the man blushes when accosted by the other; that is to say, the man is more timid in America than the woman. The Frenchman regards this *naivete* as an irresistible charm; the American seems to admire *aplomb*—the eyes which look boldly into his, and the tongue which answers him with ease and glibness. The Gallic matron affirms that she has the manners of a married woman. She goes to theatres where her daughter is never permitted to go, and reads novels that are only allowed to the French woman with a husband; orders her raiment without comment from her mother, and receives men visitors alone, and talks to them by the hour; walks fearlessly down the Champs Elysees unattended, attired in striking colors, engages her own cab, and generally manages all affairs relating to herself. Most remarkable of all, she selects her own husband.

The French mother emphatically condemns this mode of bringing up the American girl. To her, the freedom of manner and independence of character are in bad taste, and apt to lead to results that may not be named. If statistical proof be submitted to her that such an education is not incompatible with morality, she will respond that it may suit the character of the American, but would never answer for the French girl. If she be frank, she will say that she would sooner see her daughter take the veil than follow the transatlantic mode of life. But this would never be said to an American—the rules of politeness forbid it; such confidences are for the ear of her own people. If asked by an American what she thinks of his young country-women, she will probably answer that they are "charming;" hence the English and American charges of insincerity usually laid upon her shoulders. She, doubtless, says to herself, "*A quoi bon?* let us live peacefully together while we can, and make each other happy." When there is a necessity for using a sharp tongue, it is hardly necessary to add, she is not behind her sisters of any other land.—*Albert Rhodes, in "The Galaxy" for May.*

### A JAPANESE PAPER FACTORY.

About two hundred miles from Yeddo is a plain twenty miles long, and varying from two to eight miles in width. Around this plain rise mountains covered with the paper mulberry trees, and in various villages upon this plain is paper manufactured. One of these manufacturers is now continuing the business which has been prosecuted in the same place by his family for not less than six hundred years. In a yard near his house a dozen boys and girls were seen by the visitor employed in peeling the mulberry branches, which had been dried and mascerated until the soft inner bark could be readily separated from the outer. These strips were then boiled in a lye formed from the ashes of rice straw ; they were afterwards beaten almost to pulp, and finally thrown into a vat of sizing made from the bark of the slippery elm and ground boiled rice. They were agitated in this mixture until the sheets could be removed singly on fine matting, to be fastened on boards and dried in the sun. The sheets are afterwards pressed in an ordinary wedge and lever press, and finally receive a finishing gloss. The establishment employed forty persons at from six to eight cents per day, and cleared about $1,000 a year.

### INFANT PRODIGIES.

I have, I trust, great tenderness for all children ; but I know I have a special place in my heart for those poor little creatures who figure in circuses and shows, or elsewhere, as " infant prodigies." Heaven help such little folk ! It was an unkind fate that did not make them common-place, stupid, happy girls like our own Fannys and Charleys and Harrys. Poor little waifs, that never know any babyhood or childhood—sad human midges, that flutter for a moment in the glare of the gaslights, and are gone. Pitiful little children, whose tender limbs and minds are so torn and strained by thoughtless task masters, that it seems scarcely a regretable thing when the circus caravan halts awhile on its route to make a little grave by the wayside.

I never witness a performance of child acrobats, or the exhibition of any forced talent, physical or mental, on the part of children, without protesting, at least in my own mind, against the blindness and cruelty of their parents or guardians, or whoever has care of them.

I saw at the theatre the other night two tiny girls, mere babies they were, doing such feats upon a bar of wood suspended from the ceiling as made my blood run cold. They were twin sisters, these mites, with that old young look on their faces which all such unfortunates have. I hardly dared glance at them, up there in the air, hanging by their feet from the swinging bar, twisting their fragile spines and distorting their poor little bodies, when they ought to have been nestled in soft blankets in a cosy chamber, with the angels that guard the sleep of little children hovering about them. I hope the father of those two babies will read and ponder this page on which I record not alone my individual protest, but the protest of hundreds of men and women who took no pleasure in that performance, but witnessed it with a pang of pity.

There is a noble "Society for the Prevention of Cruelty to Dumb Animals." There ought to be a Society for the Prevention of Cruelty to Little Children; and a certain influential gentleman, who does some things well and other things very badly, ought to attend to it. The name of this gentleman is Mr. Public Opinion.—*T. B. Aldrich.*

### SCIENTIFIC MEMORANDA.

M. Bert states that compressed oxygen is not only destructive to animal life, but that it also hinders the germination of seeds, the putrefaction of fragments of muscle, the change of starch into sugar by saliva, and the development of *mycoderma aceti.*

If castor-oil is mixed with glycerine and a few drops of oil of cinnamon added, the taste of the castor-oil can scarcely be recognized.

Dr. Woods relates the following circumstance, which appears to show that sometimes, at least, malarial poison is to be found in water, and not in the air: Two ships were dispatched simultaneously with troops from Algeria to France, both under similar circumstances, excepting that the supply of water had been drawn, in one case, from the low, marshy lands where ague was prevalent, whilst the other ship had taken water from a locality situated at a greater elevation, and where the disease was unknown. The passengers on the first transport were generally seized with remittent fever, whereas no case of illness occurred on the other vessel.

### THE GREAT TEMPERANCE MOVEMENT.

For years, and years, and weary, suffering years, multiplied into decades, have the women of America waited to see that traffic destroyed, which annually sends sixty thousand of their sons, brothers, fathers and husbands into the drunkard's grave. They have been impoverished, disgraced, tortured in mind and body, beaten, murdered. Under the impulse of maddening liquors the hands that were pledged before heaven to provide for and protect them, have withdrawn from them the means of life, or smitten them in the dust. Sons whom they have nursed upon their bosoms with tenderest love and countless prayers, have grown into beasts, of whom they were afraid, or have sunk into helpless and pitiful slavery. They have been compelled to cover their eyes with shame in the presence of fathers whom it would have been bliss for them to hold in honor. They have been compelled to bear children to men whose habits had unfitted them for parentage—children not only tainted by disease, but endowed with debased appetites. They have seen themselves and their precious families thrust into social degradation, and cut off forever from all desirable life by the vice of the men they loved. What the women of this country have suffered from drunkenness, no mind, however sympathetic, can measure, and no pen, however graphic, can describe. It has been the unfathomable black gulf into which infatuated multitudes of men have thrown their fortunes, their health, and their industry, and out of which have come only—in fire and stench—dishonor, disease, crime, misery, despair and death. It is the abomination of abominations, the curse of curses, the hell of hells!

For weary, despairing years, they have waited to see the reform that should protect them from further harm. They have listened to lectures, they have signed pledges, they have encouraged temperance societies, they have asked for and secured legislation, and all to no practical good end. The politicians have played them false; the officers of the law are unfaithful; the government revenue thrives on the thriftiness of their curse; multitudes of the clergy are not only apathetic in their pulpits, but self-indulgent in their social habits; newspapers do not help, but rather hinder them; the liquor interest, armed with the money that should have bought them prosperity, organizes against them; fashion opposes them; a million fierce appetites are arrayed against them, and, losing all faith in men, what can they do? There is but one thing for them to do. There is but one direction in which they can look, and this is upward! The women's temperance movement, begun and carried on by prayer, is as natural in its birth and growth as the oak that springs from the acorn. If God and the God-like element in woman cannot help, there is no help. If the pulpit, the press, the politicians, the reformers, the law, cannot bring reform, who is left to do it but God and the women? We bow to this movement with reverence. We do not stop to question methods; we do not pause to query about permanent results. We simply say to the glorious women engaged in this marvelous crusade: "MAY GOD HELP AND PROSPER YOU, AND GIVE YOU THE DESIRE OF YOUR HEARTS IN THE FRUIT OF YOUR LABORS!"

It becomes men to be either humbly helpful or dumb. We who have dallied with this question; we who have dispassionately drawn the line between temperance and total abstinence; we who have deplored drunkenness with wine glasses in our hands; we who have consented to involve a great moral reform with politics; we who have been politically afraid of the power of the brutal element associated with the liquor traffic; we who have split hairs in our discussions of public policy; we who have given social sanction to habits that in the great cities have made drunkards of even the women themselves, and led their sons and ours into a dissolute life; we who have shown either our unwillingness or our impotence to save the country from the gulf that yawns before it, can only step aside with shame-faced humility while the great crusade goes on, or heartily give to it our approval and our aid.

This is not a crusade of professional agitators, clamoring for an abstract right, but an enterprise of suffering, pure and devoted women, laboring for the overthrow of a concrete wrong. It is no pleasant, holiday business in which these women are engaged, but one of self-denying hardship, pregnant in every part with a sense of duty. It is the offspring of a grand religious impulse which gives to our time its one superb touch of heroism, and redeems it from its political debasement and the degradation of its materialism. It is a shame to manhood that it is necessary; it is a glory to womanhood that it is possible.

If the experience of the last century has demonstrated anything, it is that total abstinence is the only ground on which any well-wisher of society can stand. The liquor traffic has been bolstered up for years, and is strong to-day, simply through influence which is deemed respectable. It must be made infamous by the combination of all the respectable elements of society against it. It must cease to be respectable to drink at all. It must cease to be respectable to rent a building in which liquors are sold. There is no practicable middle ground. So long as men drink temperately, men will drink intemperately, whether it ought to be otherwise or not; and it is with reference to the development of a healthy public opinion on this subject that we particularly rejoice in the women's crusade. Our own vision is so blinded and perverted that we can only see the deformity of the monster which oppresses us through woman's eyes, uplifted in prayer, tearful in shame and suffering, or bright in triumph as the strongholds of her life-long enemy fall before her.—*Dr. J. G. Holland, in "Scribner's" for May.*

### HOME-MADE.

When the words "home-made" are applied to persons or things, they imply that the same are fair, sound and good. Nothing can emanate from a well ordered, affectionate and genuine minded home circle without bearing the evidences of the grace and goodness of the place where it was formed. So much has been said, and written and sung about home, that its praise sounds dull, excepting to those who encounter the shallow counterfeits of ideal men and women in boarding houses, and live on the bakers in hotels and restaurants. To persons cast about from year to year, the home theme is never tiresome. Anything home-like, that comes from the sacred surroundings of a united family, will be recognized and hailed, as the flash from a diamond is known and appreciated as the light from the true gem.

Home-made people are the ones invariably singled out as the most excellent part of any society. Fashionable folks, or the scions of society, seem poor indeed when compared with characters disciplined and molded in pure, unworldly homes. How does the "girl of the period," whom we everywhere recognize as the type of the New York fashionable young lady, appear when seen side by side with the modest, unselfish, affectionate and capable girl, whose thoughts are more centered in home duties than in furbelows and "latest styles," and whose bright blushes are more beautiful ornaments than the jewels coveted and cried for by the once indulged society belle? How does the elegant dowager, who lives at one or another of our great hotels, or, perchance, is the nominal head of an establishment where a housekeeper keeps the wheels in motion—how does this woman, the leader of the *beau monde*, who lives for fashion and society, and every heartless sham, compare with the sweet home-made matron, whose mind is occupied with household tasks, and whose heart bestows rich love on husband and children—who rises early and lets in the sunshine to the parlor, and sees every speck of dust shaken out; who picks the dew-laden nosegay for the breakfast table; who knows that the home-made loaf is light and delicious, and the coffee *just right?* Her home and her own fair disposition greet each inmate with so fresh a "Good morning," that its blessing seems to follow the hours of the day.

The gracefully uttered "Good evening," wished in the ball room, or spoken in fashionable scenes, accompanied with charming gestures, may be fascinating and admirable, but choose between that and the tender home-made "Good night," said by a dear mother or gentle sister at your bedside, while she tucks you warmly in—which makes the heart the gladdest? The beautiful melody of the opera casts a magic spell over the richly clothed audience, who listen entranced to the prima donna's brilliant tones; but mark you the lullaby song of home—the tune to which baby is "byed" away to sleep at night—can artistic music clasp about your soul as will that sweet, simple, cradle song?

There is home-made food, of which we all are so fond, and with which the most complicated dishes of the fashionable caterer cannot compare in excellence. Was ever such cook known as our own grandmother, and next to her, our mother? The toast, tea, preserves and gruel made at home when we were children—have we ever forgotten their relish, or when ill, have we ceased to yearn for these home-made tit-bits of long ago? The pies, cakes and biscuits of home manufacture—where is the baker who can make any half so good?

There are certain things to which the appellation of home-made is not agreeable. Home-made looking bonnets or dresses, or coats or trowsers, mean that these articles appear dowdy or botched, and not as if made by clever hands. There may not be any style about home-made clothes, but it may be said for the consolation of those who cannot afford to patronize milliners and mantua makers, that there is a respectable air of economy in a home-made garment which is always attractive to superior minds. Every one appreciates the beauty of home-made underwear, now that so many buy the machine work which fills the leading stores. Compare the fine hand-sewed "pillow case," with its neatly stitched yoke, its evenly laid gathers, and whipped linen frill, with those of the same shape found in the best shops.

There are numbers of people who have homes who are never *made* or influenced by them; there are plenty of so-called homes which never produce anything home-made. In these sham homes the mothers are sure to be found "out of tune." The father is a club man. The daughters are selfish and lackadaisical—good for nothing in the sick room—would never offer to bathe an aching head, and did they attempt to perform this grateful act, would not do it acceptably. They are not good for much anyway, unless it is to be married, and make some man miserable, and let their babies die with cholera infantum—another name for want of proper care.

"Home-made" is a good motto—a dear motto. Let it be stamped on every person and thing that crosses our thresholds, and it shall bring "Peace to this house."

### A SENSIBLE LETTER.

Mme. Hyacinthe Loyson writes for the benefit of the lady crusaders to good purpose. She says:

"The great American malady is the malady of the stomach. Conscientious people become dyspeptics; non-conscientious people become drinkers. The appetite for drink is not necessarily made by drinking, but in nine cases out of ten, by the use of coffee, tea, pepper, pickles, mustard, spices, too much salt, hot bread and pastry, raw meat and grease, and above all, tobacco. The cry of a depraved appetite, an inflamed stomach, is always for something stronger. The use of soup, milk and salad, prepared with good oil, should be cultivated. Instead of rye for whiskey, raise grapes, that pure native wine may be used when needed. Distilleries are slaughter houses of men, and should be dealt with as such; and money gained from selling liquor is blood money, and should never touch the palm of an honest man."

### FASHION IN HAVANA.

Riding on the Paseo de Tacon, a beautiful broad street with a noble fountain and two statues as turning places for the carriages, I saw something of the freedom of the women in certain respects. While the rules of etiquette are very strict in many ways, in other respects they are allowed liberties of dress and demeanor that surprise us: I think it is as you tolerate certain follies in children. For instance, they break out in violent color in their costumes, which strongly contrast with their dark hair and eyes. Pink and scarlet toilets are the rage; bare heads, necks and arms, the style. As you ride up and down the Paseo, which is the great course for carriages, you pass and repass many handsome equipages, drawn by English or American horses, the beasts of the country being very slink and disconsolate, as they well may be from the ill usage that is heaped upon them. These carriages are all open, and oftener they are the English victoria with two seats, or the Spanish volante, which is a low chaise, often painted yellow, hung on two long, slender thills, between which one horse trots, while another horse, attached by outside traces, with a mounted groom, in boots, cocked hat and a livery, gallops by its side. The top of the volante flops down over the passengers like an old-fashioned calash, and you can only catch glimpses of the Cuban aristocrats who cling to this ungainly national vehicle. But you can discover the uncovered heads, and the elaborately dressed hair, and the nude necks and arms, and the bright pink and buff satin dresses that overflow the sides of the volante. Ladies ride alone without any cavaliers, and receive any compliments which strangers from the sidewalk may pay them with great *sang froid,* if not with gracious smiles.

The "circles of conversation" indicate the formal relations between men and women. These arrangements consist of ten chairs placed opposite to each other, with a chair between at the head, and a sofa at the foot, where the mother sits. The father takes the chair at the head, and the males sit on one side and the females on the other, like a Quaker meeting; only they all talk, and very fast, so that the effect of this cross speech or fire is very bewildering, as each person speaks to his opposite neighbor instead of the one at his side. It is thought very improper if a lady and gentleman sit side by side upon a sofa or in two chairs. Indeed, a Spaniard rises as if insulted if an English or American lady should take a seat near him, for fear of interrupting the conversation of others. Political conversation was in bad taste, and only by accident could a foreigner learn anything of the history and present attitude of the two parties who are involved in a ceaseless struggle for principles and power.—*Mrs. Ford, in "The Galaxy" for May.*

### CLEANING BOTTLES.

A GENTLEMAN having spent sixty thousand dollars in the erection of a country seat, set himself down with his young wife and little child to enjoy himself.

In the course of a few months cider making came around, and some old bottles were filled with cider. Later on a bottle was placed on the table and drank. In a short time the husband was taken with vomiting and died. The wife lingered two years and died also.

One of the bottles was found to have corrosive sublimate at the bottom. Some persons clean bottles by putting in some small shot and shaking them around. Water—hot water—dissolves lead to a certain extent, and a film of this lead attaches itself to the sides of the bottle so closely that the shaking or rinsing with water does not detach it, and it remains to be dissolved by any liquid which has the least sourness in it, and if drank, lead poison is the result; sometimes a shot becomes wedged in at the bottom of a bottle, to be dissolved by wine or cider. There's safety only in two precautions. When once a poison has been put into a bottle, break it to pieces as soon as used. Second, wash every bottle, as soon as emptied, with warm water and wood ashes, or salaratus, and put the bottles away, mouth open and downward.

### HOMAGE.

I.

White daisies on the meadow green
  Present thy beauteous form to me ;
Peaceful and joyful these are seen,
  And peace and joy encompass thee.
I watch them where they dance and shine,
And love them—for their beauty's thine.

II.

Red roses o'er the woodland brook
  Remember me thy lovely face ;
So blushing and so fresh its look,
  So wild and shy its radiant grace.
I kiss them in their coy retreat,
And think of lips more soft and sweet.

III.

Gold arrows of the merry morn
  Shot swiftly over Eastern seas,
Gold tassels of the bending corn
  That ripple in the August breeze,
Thy wildering smile, thy glorious hair,
And all thy power and state declare.

IV.

White, red, and gold—the awful crown
  Of virtue and of beauty, too !
From what a height those eyes look down
  On him who proudly dares to sue.
Yet, free from self as God from sin
Is love that loves nor asks to win.

V.

Let me but love thee in the flower,
  The waving grass, the dancing wave,
The fragrant pomp of garden bower,
  The violet on the nameless grave.
Sweet dreams by night, sweet thoughts by day,
And time shall tire ere love decay.

VI.

Let me but love thee in the glow
  When morning on the ocean shines,
Or in the mighty winds that blow,
  Snow-laden through the mountain pines—
In all that's fair, or grand, or dread—
And all shall die, ere love be dead.

*William Winter, in "The Galaxy" for May.*

### TIM McGOWAN.

THIS gallant fellow lost his life in the Mexican war. He had lost an arm when a boy by having the limb crushed under the wheel of a jaunting car, in the "ould counthry." His surviving brother, Dennis, never ceases boasting of Tim's exploits. In a Moyamensing bar room, the other evening, Dennis began on the old theme of the Mexican war, dwelling with particular emphasis on the heroic deeds of his deceased relative:

"Och, murther, but ye ought to have seen Tim at Rye-sack-a-dollar-pole-me (meaning Resaca de la Palma). He caught two Mexican blackguards by the cuffs of their necks and kilt them both as dead as herrin's by knocking their heads together."

"How could that be," said a listener, "when your brother had but one arm?"

"Bless yer sowl," answered Dennis, "one arm had he! That's true enough for ye; but then, ye see, Tim *forgot* all about that when he got into a fight!"

### A GOOD RULE.

'TIS well to walk with a cheerful heart,
  Wherever our fortunes call,
With a friendly glance and open hand,
  And a gentle word for all.

Since Life is a thorny and difficult path,
  Where toil is the portion of man,
We all should endeavor, while passing along,
  To make it as smooth as we can.

